# Patient Preferences for Attributes of Chemotherapy for Lung Cancer: Discrete Choice Experiment Study in Japan

**DOI:** 10.3389/fphar.2021.697711

**Published:** 2021-07-20

**Authors:** Yasuo Sugitani, Kyoko Ito, Shunsuke Ono

**Affiliations:** ^1^ Biometrics Department, Chugai Pharmaceutical Co., Ltd., Tokyo, Japan; ^2^ Laboratory of Pharmaceutical Regulation and Sciences, Graduate School of Pharmaceutical Sciences, The University of Tokyo, Tokyo, Japan; ^3^ Sustainability Department, Chugai Pharmaceutical Co., Ltd., Tokyo, Japan

**Keywords:** patient centricity, Japanese patient preferences, lung cancer, discrete choice experiment, conjoint analysis, hierarchical bayes model

## Abstract

Our study objective was to determine lung cancer chemotherapy attributes that are important to patients in Japan. A discrete choice experiment survey in an anonymous web-based questionnaire format with a reward was completed by 200 lung cancer patients in Japan from November 25, 2019, to November 27, 2019. The relative importance of patient preferences for each attribute was estimated using a conditional logit model. A hierarchical Bayesian logit model was also used to estimate the impact of each demographic characteristic on the relative importance of each attribute. Of the 200 respondents, 191 with consistent responses were included in the analysis. In their preference, overall survival was the most important, followed by diarrhea, nausea, rash, bone marrow suppression (BMS), progression-free survival, fatigue, interstitial lung disease, frequency of administration, and duration of administration. The preferences were influenced by demographic characteristics (e.g., gender and age) and disease background (e.g., cancer type and stage). Interestingly, the experience of cancer drug therapies and adverse events had a substantial impact on the hypothetical drug preferences. For the Japanese lung cancer patients, improved survival was the most important attribute that influenced their preference for chemotherapy, followed by adverse events, including diarrhea, nausea, rash, and BMS. The preferences varied depending on the patient’s demographic and experience. As drug attributes can affect patient preferences, pharmaceutical companies should be aware of the patient preferences and develop drugs that respond to segmented market needs.

## Introduction

With the growing number of available drug therapies, patients have more treatment choices. Healthcare providers should have an accurate and comprehensive understanding of patient preferences and needs as the treatment value should be derived from its significance to the patient. Regulatory agencies in the United States and European countries and some private organizations advocate for patient centricity, patient preferences, and dialog (Muhlbacher et al., 2016; Myeloma 2019; DIA 2021; EMA 2021; FDA 2021). Therefore, eliciting patient preference for treatment attributes is becoming essential for drug development.

Previous studies have demonstrated that treatment selection based on patient preferences has a significant impact on adherence (Von Korff et al., 2003; Wilson et al., 2010). The importance of patient preferences in decision-making processes, including benefit-risk assessment (Coplan et al., 2011; MDIC 2015; Schnipper et al., 2015; ICH 2016; FDA 2018; Postmus et al., 2018; Van Overbeeke et al., 2019), is underscored by the emergence of conflicts between physicians and patients when they differ in their preferred treatment choices (Mokhles et al., 2018; Valenti et al., 2020).

Of all cancer types, lung cancer has the highest prevalence worldwide. Approximately 2.2 million new lung cancer cases and 1.8 million deaths were reported in 2020, accounting for 18% of all cancer-related deaths (Sung et al., 2021). In Japan, lung cancer has the third-highest prevalence among cancers, with 124,510 new cases in 2017, according to the most recent cancer statistics (Foundation for Promotion of Cancer Research, 2021). In most countries, five-year survival rate for lung cancer patients is 10–19% during 2010 through 2014. (Allemani et al., 2018). Of all cancer deaths, lung cancer accounts for the highest mortality in Japan, with 75,394 deaths in 2019, and the five-year relative survival rate of lung cancer was 34.9%, the third worst among cancers of any major site in Japan.

Due to the poor prognosis of lung cancer, the choice of therapy is essential. The various lung cancer treatment options according to recent guidelines in the United States, EU, and Japan include cytotoxic anticancer drugs (e.g., cisplatin and docetaxel), molecularly targeted drugs (e.g., bevacizumab and crizotinib), and immune checkpoint inhibitors (e.g., pembrolizumab and atezolizumab) (Hanna et al., 2017; Planchard et al., 2018; Japan Lung Cancer Society 2019). For traditional cytotoxic chemotherapy, adverse events and their impact on antitumor efficacy and survival are considered in treatment decisions (Blinman et al., 2010; Blinman et al., 2011; Benz et al., 2019).

Preference studies on treatment attributes and their impact on patients’ value are gaining prominence in many countries (Blinman et al., 2010; Schmidt et al., 2016). A literature review by Blinman et al. (2010) suggests that overall survival (OS) is an important attribute for patients in treatment selection. It also showed that the preferred treatment choices depend on cancer metastases/localization, drug toxicities, and the region of study (e.g., North America vs. Japan) and that the level of dependency varies according to age, presence of dependent families, educational attainment of at least a college degree, and baseline quality of life. Schmidt et al. (2016) noted that patient preferences do not uniformly depend on patient demographic characteristics, such as age. Additionally, Sugitani et al. (2020) suggested that lung cancer patients emphasize efficacy attributes, especially OS, relative to safety and clinical utility attributes (e.g., route of administration).

Quantitative analysis of lung cancer patients to identify the demographic characteristics associated with their preferences is often conducted by latent class analysis or subgroup analysis (Hirose et al., 2005; Muhlbacher and Bethge 2015; Schmidt et al., 2017). The hierarchical Bayesian model, which is based on the hypothetical model, has been used for patients with diabetes (Flood et al., 2017) and multiple sclerosis (Garcia-Dominguez et al., 2016), but not for patients with lung cancer.

Among Japanese patients with lung cancer, only three preference studies have been conducted. Hotta et al. (2010) focused on shared decision-making, while Hirose et al. (2005; 2009) examined the acceptability of intensive treatment using the standard gamble method; lung cancer patients younger than 70 years of age and patients without cancer younger than 65 years of age were more likely to tolerate intensive treatment. No studies have investigated both benefit and risk attributes simultaneously in Japanese patients using rigorous methods such as discrete choice experiment (DCE).

## Materials and Methods

### Preference Methods and Development of Survey

We investigated patient preferences for the attributes reflecting the benefits, risks, and utility of cancer drug treatment. We conducted a conjoint analysis (or a DCE, specifically) as it has previously been used to study patient preference in the healthcare field (Ryan and Gerard 2003; De Bekker-Grob et al., 2012; Hauber et al., 2016) and is less burdensome for respondents. This study was conducted according to the ISPOR’s checklist for the implementation of conjoint analysis (Bridges et al., 2011). First, the attributes to be used in DCE were selected based on the lung cancer treatment guidelines by the American Society of Clinical Oncology (Hanna et al., 2017), the European Society for Medical Oncology (Planchard et al., 2018), and the Japan Lung Cancer Society (2019). OS and progression-free survival (PFS) were selected as benefit attributes as they are indicators of efficacy. In contrast, the risk attributes were diarrhea, nausea or vomiting, fatigue or general malaise, and rash; they occurred in more than 10% of patients in pivotal clinical trials as side effects of more than half of the anticancer drugs approved in Japan. Interstitial lung disease (ILD) and bone marrow suppression (BMS) were also selected, which have attracted attention, especially in Japan. The frequency and duration of administration were included because most anticancer drugs, except molecular targeted drugs, are intravenous, and these attributes vary significantly between drugs.

The level of each attribute was based on the best and worst levels of the drugs listed in the guidelines. The risk levels were described in terms of incidence rate, and the severity of each risk was assumed to be fixed, corresponding to the drug descriptions listed in the guidelines.

After creating hypothetical drug profiles comprising the aforementioned attributes and levels, a 24-question discrete choice questionnaire was first piloted anonymously to pharmaceutical experts of the two institutions to which the authors belong. We reduced the number of discrete choice questions as some respondents felt burdened. The questionnaire was also reviewed by the Lung Cancer Patient Network ONE STEP, a Japanese patient organization, to ensure that the questions, including the inquiries of the patient’s background, met ethical requirements and were easy to understand and the selected drug profiles were appropriate. Based on the suggestions by the patients’ association, we simplified the explanations and provided examples of questions in the consent statement.

To reduce the respondent burden, we used a two-choice format, with no option of choosing neither. The number of levels for each attribute was minimized, with three OS levels and two for the other characteristics (Table 1).

**TABLE 1:** Attributes and levels for the discrete choice experiment.

Attribute	Description	Level		
OS	Half of the people whose cancer status is stage 3 or higher and who use the medicine are expected to live longer than this period of time.	20 months	15 months	10 months
PFS	Half of the people whose cancer status is stage 3 or higher and who use the medicine are expected to have their cancer not to progress beyond this time and to be stable.	9 months	4 months	
Diarrhea	This percentage of people who use the medication are expected to have about a week of diarrhea with about five to six bowel movements every day.	50%	100%	
Nausea or vomiting	This percentage of people who use the medication are expected to have 3–4 days of nausea with obvious weight loss and loss of appetite, or vomiting about 4 times a day.	10%	50%	
Fatigue or general malaise	This percentage of people who use the medication are expected to have 3–4 days of fatigue and sluggishness that does not recover after a break and interferes with activities of daily living such as cooking, washing, and cleaning.	10%	30%	
Rash	This percentage of people who use the medication are expected to develop raised or pus-filled pimples on 10% to 30% of their body, such as the face, chest, and back, which will interfere with daily activities of living such as cooking, washing, and cleaning for about 2–3 weeks.	10%	80%	
Interstitial lung disease	This percentage of people who use the medication are expected to develop severe inflammation of the lungs, requiring hospitalization, treatment, and oxygen inhalation, which will interfere with activities of daily living such as eating, dressing, and toileting.	0%	5%	
Bone marrow suppression	This percentage of people who use the medication are expected to have a severe decrease in white blood cells, hemoglobin, neutrophils, and platelets, which makes them more susceptible to various infections, makes them tired and dizzy due to anemia, makes them bleed easily and difficult to stop bleeding, and requires hospitalization and treatment for about a week.	0%	20%	
Frequency of administration	To use the medication, going to the hospital at this frequency will be needed.	every week	every 3 weeks	
Duration of administration	To get the medication in by injection, staying in bed for this long will be needed.	30 min	180 min	

OS: Overall Survival, PFS: Progression-Free Survival.

**FIGURE 1:**
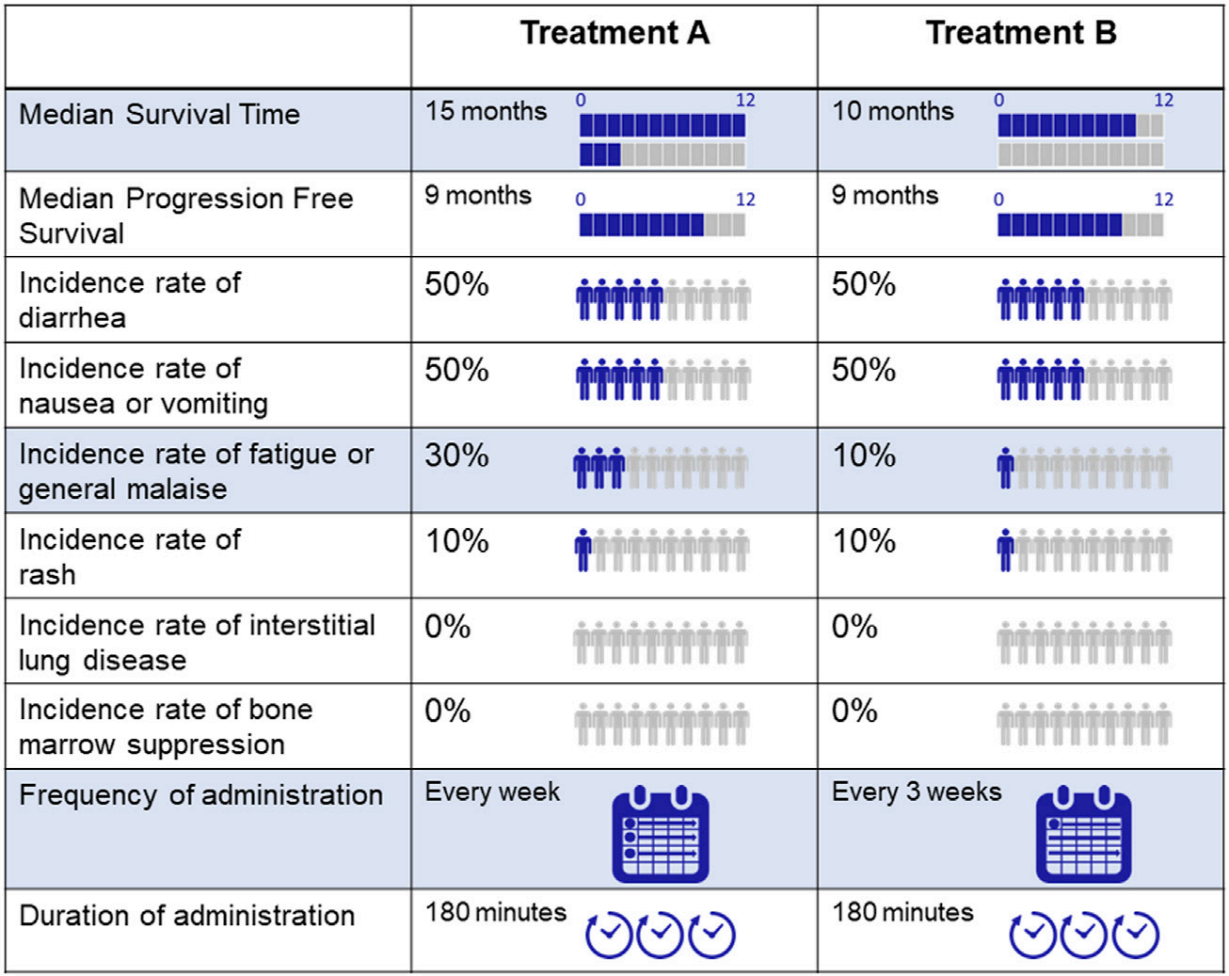
An example of the questionnaire.

In order to consider the efficiency of the estimation, based on the D-optimal design, the number of profiles were 48. The less than 48 profiles dropped the D-efficiency significantly. We created a questionnaire in Japanese comprising comparative combinations of profiles with the same attribute levels. Two sets of 14 questions were prepared, including four questions with one attribute difference between two profiles, to check the respondent’s reliability. The respondents answered the 14 questions from a randomly assigned set. We did not evaluate the strength of the preference or the level of confidence in the response; we simply queried the preferred treatment. An example of the questionnaire is shown in Figure 1.

Based on previous literature (Sugitani et al., 2020), we collected the following demographic characteristics of the respondents: gender, age, province of residence, working status, living situation, dependents, education level, annual income, type of lung cancer, stage of lung cancer, systemic symptoms of lung cancer: Eastern Cooperative Oncology Group performance status (ECOG PS), smoking history, the experience of surgery for cancer, the experience of radiotherapy for cancer, the experience of anticancer drugs, and experience of adverse events of anticancer drugs. Unknown or unwilling to answer some of these questions about demographic characteristics was also indicated as an option.

### Recruitment

An anonymous web-based questionnaire with a reward was administered from November 25, 2019, to November 27, 2019. Out of the 448 lung cancer patients in the panel held by Rakuten Insight, Inc., 200 people were enrolled in our study.

The minimum sample size as calculated by the Johnson and Orme method (Orme 1998; Johnson and Orme 2003) for conjoint analysis was 54 patients [= (500 * 3)/(2 * 14)]. Johnson et al. (2013) reported that SD began to settle at around 150 when using the conditional logit model. Therefore, based on these rationales and the possibility of a certain number of invalid responses due to lack of reliability, the sample size was set at 200 patients.

Prospective participants were informed that they needed to be lung cancer patients to participate in the study. Lung cancer diagnosis was based on the participant’s self-report, and no medical diagnosis was made to confirm the condition.

### Statistical Analysis

The relative importance of patient preferences for each attribute was estimated using a conditional logit model (see Supplementary Material 1 for equations). We excluded respondents who chose the same option (i.e., “Treatment A” or “Treatment B”) for all questions. In addition, we conducted sensitivity analyses excluding the respondents who did not choose the “correct” answer to a set of questions for which one treatment dominates the other [i.e., one attribute level of Treatment A (or B) was better than the attribute level of Treatment B (or A), and the other attribute levels were the same]. For the subgroup analysis, the relative importance of a patient preference for each attribute was estimated using a conditional logit model, focusing only on the population of each demographic characteristic. For the relative importance results, the absolute values indicate the importance of the attribute, with a positive sign indicating a more favorable impact and a negative sign indicating a less favorable impact.

A hierarchical Bayesian logit model was used to consider multiple demographic characteristics, and the posterior distribution of the impact of each demographic characteristic on the relative importance of each attribute was estimated (Supplementary Material 1 for equations). The prior distribution and each parameter followed the default settings, and the number of simulations was set to 110,000. The first 10,000 simulations were truncated as burn-in, and the posterior distribution was generated by picking up the result once every 100 times. Missing demographic characteristics (i.e., unknown or unwilling to answer) were imputed by the mode of values. Sensitivity analysis was performed to exclude missing respondents. All model analyses were performed using R version 3.4.3. The library used for the analysis was “conjoint” (version 1.41) and “bayesm” (version 3.1.1).

### Ethics

The study was approved by an ethics committee registered with the Ministry of Health, Labour and Welfare (registration No. 11001059), and the study registration was published in the University Hospital Medical Information Network (UMIN) as study ID: UMIN000039087. We obtained prior written consent from the respondents for analysis and publication of the results.

## Results

### Study Population

Of the total 200 respondents, 191 were included in the subsequent analysis, excluding the nine unreliable respondents who chose the same option for all questions. The patient background data is shown in Table 2. The proportion of males was 82.7% (158/191), which was considerably higher than in previous studies (Sugitani et al., 2020). The mean age was 63.3 years, similar to that reported in previous studies (Sugitani et al., 2020). Most of the patients were in the early lung cancer stage, with 82 patients (42.9%) in Stage I.

**TABLE 2:** Demographic characteristics of respondents.

Characeristics	
Gender, n (%)	N=191
Male	158 (82.7%)
Female	33 (17.3%)
Age in years, mean (SD)	63.3 (9.4)
Age group, n (%)	
30–39	4 (2.1%)
40–49	12 (6.3%)
50–59	38 (19.9%)
60–69	75 (39.3%)
70–79	62 (32.5%)
Geographic area	
East Japan	110 (57.6%)
West Japan	81 (42.4%)
Type, *n* (%)	
NSCLC	172 (90.1%)
SCLC	18 (9.4%)
Unknown	1 (0.5%)
Not answered	0 (0.0%)
Stage, *n* (%)	
I	82 (42.9%)
II	22 (11.5%)
III	22 (11.5%)
IV	33 (17.3%)
Unknown	31 (16.2%)
Not answered	1 (0.5%)
Education level, *n* (%)	
Elementary and junior high school	5 (2.6%)
Senior high school	54 (28.3%)
Junior college and vocational school	26 (13.6%)
University degree	99 (51.8%)
Postgraduate degree	7 (3.7%)
Not answered	0 (0.0%)
Working status, *n* (%)	
Not working	98 (51.3%)
Part-time working	22 (11.5%)
Full-time working	70 (36.6%)
Not answered	1 (0.5%)
Living situation, *n* (%)	
Live alone	27 (14.1%)
Live with others	163 (85.3%)
Not answered	1 (0.5%)
Dependents, *n* (%)	
None	47 (24.6%)
Spouse	98 (51.3%)
Spouse and children, or just children	40 (20.9%)
Other	6 (3.1%)
Not answered	0 (0.0%)
Annual income	
−1 million yen	22 (11.5%)
1–4 million yen	79 (41.4%)
4–6 million yen	31 (16.2%)
6–8 million yen	19 (9.9%)
8–10 million yen	13 (6.8%)
10+ million yen	12 (6.3%)
Not answered	15 (7.9%)
ECOG PS, n (%)	
0	126 (66.0%)
1	59 (30.9%)
2	3 (1.6%)
3	3 (1.6%)
4	0 (0.0%)
Not answered	0 (0.0%)
Smoking history, n (%)	
Never	51 (26.7%)
less than 1 year	2 (1.0%)
1–5 years	9 (4.7%)
5–10 years	11 (5.8%)
10+ years	118 (61.8%)
Not answered	0 (0.0%)
Experience of surgery for cancer	
No	33 (17.3%)
Yes	158 (82.7%)
Not answered	0 (0.0%)
Experience of radiotherapy for cancer	
No	142 (74.3%)
Yes	49 (25.7%)
Not answered	0 (0.0%)
Experience of anticancer drug	
No	76 (39.8%)
Yes	115 (60.2%)
Not answered	0 (0.0%)
Experience of adverse events by anticancer drug	
No	101 (52.9%)
Yes	89 (46.6%)
Not answered	1 (0.5%)

ECOG PS: Eastern Cooperative Oncology Group performance status, NSCLC: Non-Small-Cell Lung Cancer, SCLC: Small-Cell Lung Cancer.

### Relative Importance of the Treatment Characteristics

**FIGURE 2:**
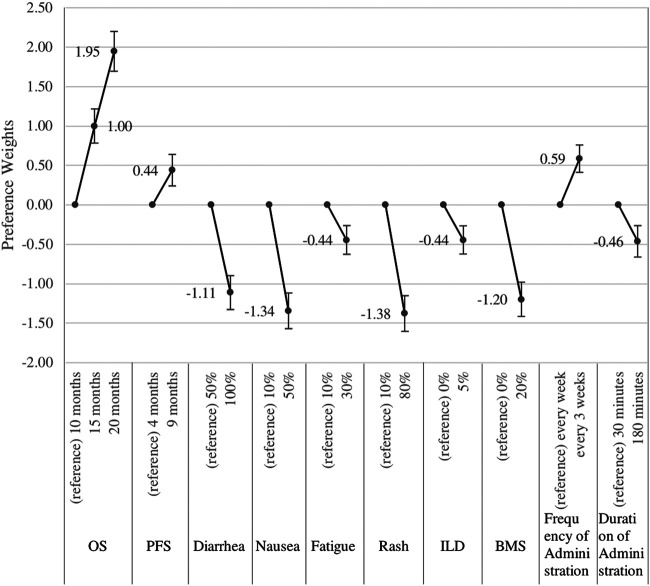
Relative importance of each attribute (*N* = 191). BMS, Bone Marrow Suppression; ILD, Interstitial Lung disease; OS, Overall Survival; PFS, Progression Free Survival.

The relative importance of patient preferences for each attribute as the main effects estimated by the conditional logit model is shown in Figure 2. An increase in OS from 10 to 20 months was the most important attribute, followed by an increase in OS from 10 to 15 months and an increase in the occurrence of diarrhea (from 50 to 100%), nausea (from 10 to 50%), rash (from 10 to 80%), and BMS (from 0 to 20%). An increase in PFS from 4 to 9 months was the least important benefit attribute, similar to an increase in fatigue (from 10 to 30%), ILD (from 0 to 5%), and frequency and duration of administration.

For sensitivity analyses, we estimated the results by excluding nine unreliable respondents who chose the same option for all questions and who did not make the correct choice in the dominated pair questions. The results of the sensitivity analyses are shown in the Supplementary Material 2. There was no significant difference in these results. Subpopulation analyses were also performed for respondents whose demographic characteristics were available. The results are shown in the Supplementary Material 3.

### Preference by Patient Demographics

Of the demographic characteristics added to the model as explanatory variables, only age was expressed on an ordinal scale for each decade, while the other items were expressed on a binary nominal scale. A chi-square test was performed to investigate the relationship between demographic characteristics (Supplementary Material 4). Based on the results, gender, age, region, type of lung cancer, stage of cancer, education level, working status, annual income, experience of anticancer drugs, and experience of adverse events of anticancer drugs were included as explanatory variables in the hierarchical Bayesian logit model. ECOG PS and experience of surgery and radiotherapy were not included in the model due to their modest relationships with cancer stage and experience of anticancer drugs, respectively. Living situation, dependents, and smoking history were not included owing to their association with gender and age. The missing demographic characteristics or those answered as “unknown” were imputed with the most frequent values. The analyses without imputation are shown in the Supplementary Material 5.

The associations between the patient demographic characteristics and the preferences for each treatment attribute are shown in Table 3. In Table 3, the finding that the category of interest is more important than the reference category is indicated by a positive coefficient for OS, PFS, and frequency and duration of administration and by a negative coefficient for diarrhea, nausea, fatigue, rash, ILD, and BMS. Compared to men, women considered increased frequency of diarrhea and nausea to be more critical. Older patients were more likely to be concerned about the increased frequency of nausea, fatigue, and rash than younger patients. Patients living in eastern Japan placed more importance on the increased frequency of nausea than those in western Japan. Compared to patients with NSCLC, SCLC patients reported less importance for prolonged OS and PFS and increased frequency of fatigue, and more importance for a prolonged duration of administration. Patients with more advanced stages (i.e., Stage II–IV) reported that prolonged OS was important. Patients with a higher education background preferred prolonged long-term survival and nausea. The working status was not significantly associated with any attribute. Patients with an annual income of more than 4 million yen showed that prolonged long-term survival was more important. Interestingly, patients who had experience with anticancer drugs considered prolonged PFS and increased frequency of rash important, but those who had previously experienced adverse events of anticancer drugs did not.

**TABLE 3:** Impact of patient demographic characteristics on preferences for each attribute.

	Intercept	Gender (ref. male)	Age (ref. 30-39)	Area (ref. East Japan)	Type (ref. NSCLC)	Stage (ref. I)	Education level (ref. lower than univ. degree)	Working status (ref. not working)	Annual income (ref. less than 4 million yen)	Exp. of anticancer drug (ref. No)	Exp. of AEs by anticancer drug (ref. No)
		female	per 10 years old	West Japan	SCLC	II-IV	univ. or higher degree	Part- or full-time working	4 million yen or more	Yes	Yes
OS (ref. 10 months)											
15 months	−0.18	−0.34	0.50	0.94	−4.13 ^−−^	3.04 ^++^	1.61 ^+^	0.03	0.50	−1.41	1.75 ^+^
20 months	−0.26	1.23	0.29	−0.64	−3.99 ^−−^	5.96 ^++^	4.66 ^++^	−0.02	3.42 ^++^	3.38 ^+^	–4.36 ^−−^
PFS (ref. 4 months)											
9 months	0.36	2.37 ^+^	−0.19	0.22	−2.42 ^−−^	1.54 ^+^	0.39	−1.06	1.18 ^−^	2.43 ^++^	−1.52
Diarrhea (ref. 50%)											
100%	0.01	−4.39 ^−−^	−0.62	−0.72	2.29 ^+^	−0.45	−2.04 ^−^	−1.47	0.83	−1.82	1.34
Nausea (ref. 10%)											
50%	0.00	−4.83 ^−−^	−1.20 ^−−^	−2.92 ^−−^	1.66	−0.78	−2.44 ^−−^	−1.33	0.29	−0.12	−1.06
Fatigue (ref. 10%)											
30%	−0.05	−0.07	−0.93 ^−−^	−1.51 ^−^	2.87 ^++^	−1.04	−0.19	0.22	0.61	−0.02	−0.40
Rash (ref. 10%)											
80%	0.25	−2.77 ^−^	−1.88 ^−−^	−1.77 ^−^	2.23 ^+^	−0.79	−1.67 ^−^	−1.98^−^	0.15	−4.57 ^−−^	4.98 ^++^
ILD (ref. 0%)											
5%	−0.23	−0.22	0.60 ^+^	1.14 ^+^	−1.24	−1.88^−^	−1.38 ^−^	0.21	0.03	−1.95	3.05 ^++^
BMS (ref. 0%)											
10%	−0.34	−1.03	−0.15	1.10	−0.97	0.11	−1.78 ^−^	−0.61	−0.46	−0.34	1.73
Frequency of Administration (ref. every week)											
every 3 weeks	0.15	1.55	0.01	0.15	−0.96	1.36 ^+^	1.22 ^+^	0.34	−0.36	−1.00	1.05
Duration of Administration (ref. 30 min)											
180 min	0.03	−0.63	−0.66	1.04	−2.84 ^−−^	0.39	1.06	−0.12	−0.10	−1.97	2.44

+: the 95% credible interval was more than zero, ++: the 99% credible interval was more than zero, -: the 95% credible interval was less than zero, --: the 99% credible interval was less than zero, BMS: Bone Marrow Suppression, exp.: experience, ILD: Interstitial Lung Disease, NSCLC: Non-Small-Cell Lung Cancer, OS: Overall Survival, PFS: Progression-Free Survival, ref.: reference, SCLC: Small-Cell Lung Cancer, univ.: university.

There was no substantial difference between these results and those of the subpopulation analysis, thus confirming the robustness of the results.

## Discussion

This is the first study to quantify preferences for the benefit and risk profile of anticancer drugs in Japanese lung cancer patients. Our results clarify that survival time is the most important attribute, followed by adverse events, PFS, and the frequency and duration of administration. Additionally, the results showed the benefit and risk attributes valued by Japanese patients in relation to demographic characteristics and experience with anticancer treatment.

With respect to the treatment benefit, Japanese patients considered OS as the most important attribute and PFS as the less important one. These preferences were consistent with those in previous studies (Blinman et al., 2010; Sugitani et al., 2020). Although some studies (Muhlbacher and Bethge 2015; Bridges et al., 2019) found PFS to be important in lung cancer, a systematic review (Raphael et al., 2019) examining PFS in various cancers found it less important than adverse events. In this study, we could not explain whether cancer progression could be related to clinical symptoms and/or OS, which could lead to less importance of PFS.

Regarding risk characteristics, patients did not consider fatigue and interstitial pneumonia important compared to the other safety attributes. Although many previous studies on lung cancer (Bridges et al., 2012; Sun et al., 2019) demonstrated the importance of fatigue, a Japanese study on other cancer types (Shiroiwa et al., 2009) showed that it was less important. In this study, we assumed a lower incidence of fatigue than in other surveys, and this might be the reason why fatigue was regarded as less important. Besides, the reason ILD was regarded as less important was because interstitial pneumonia is an adverse event unfamiliar to patients and was assumed to have a low occurrence rate in this study. Patients’ evaluation of ILD has not been reported in previous studies. Concerning other safety attributes, the importance of frequency and duration of administration was not recognized in comparison with other safety attributes; the preference for these attributes in lung cancer patients has not been reported previously. Similarly, the route of administration was not an important attribute. The low preference for attributes related to drug use (i.e., attributes other than efficacy and safety) in the current study is consistent with previous studies on lung cancer patients (Bridges et al., 2019; Sun et al., 2019) and ovarian cancer patients (Havrilesky et al., 2014).

Our regression results showed that preferences for treatment attributes are influenced by patient demographic characteristics (Table 3). Compared to men, women were more sensitive to the level of some safety attributes, including frequency of diarrhea and nausea. Our results are comparable to those reported in a study on acute myeloid leukemia (Richardson et al., 2020), where women tended to focus more on adverse events than on efficacy. The tendency for older patients to focus on adverse events such as rash was similar to that reported in previous studies on lung cancer (Hirose et al., 2005; Girones et al., 2012; Sun et al., 2019).

In this study, we reported varied importance of nausea depending on where the respondents live. However, such findings are difficult to interpret considering the lack of previous reports showing such regional disparities in the preferences of lung cancer patients. The number of physicians per population is higher in western Japan than in eastern Japan (Ministry of Health, Labour and Welfare 2018), and the per capita cost of medical care is also higher in western Japan (Ministry of Health, Labour and Welfare 2017). Although regional healthcare settings are associated with treatment access, it is difficult to ascribe the patients’ preferences for side effects such as nausea to such regional differences.

SCLC accounts for about 13–15% of lung cancers worldwide (Govindan et al., 2006; Nicholson et al., 2016; American Cancer Society 2020). Our sample had only 18 patients with SCLC, and the results may not be appropriate for generalization. The impact of cancer stage on preference was similar to that observed in previous studies (Sun et al., 2019); the importance of PFS increased with cancer progression. It was reasonable to consider survival more important for the patients with a worse prognosis.

Patients with higher education and/or income regarded OS as more important. No previous studies have shown a similar relationship between OS and education or income. A study on lung cancer (Muhlbacher and Bethge 2015) that included only PFS as the benefit attribute showed a tendency for the more educated group to focus on the benefits. A study on colorectal cancer (Fu et al., 2016) reported that higher education and income are associated with a higher tolerance for adverse events, implying that patients with these characteristics place more emphasis on efficacy.

PFS was more important in patients who had experience with anticancer drugs. This is possibly because most patients with no experience of anticancer drugs have experience of surgery; thus, they have little concern for tumor shrinkage or progression. In contrast, patients with experience of anticancer drugs focus on tumor shrinkage or progression as part of drug response. Patients with prior experience of adverse events of anticancer drugs understood the importance of the benefits and adverse events. Our results showed that the importance of long-term OS, rash, and ILD was lower in such patient groups. The low importance of these two side effects may be due to both their severity and frequency; the rash may be experienced by many respondents but is relatively less severe, while ILD, if it occurs, is severe but less frequent, and few respondents experienced it.

Our results underscore the importance of considering several demographic characteristics in the models to better estimate patient preferences. Based on the results from the hierarchical Bayesian model and the subpopulation analysis, we believe that gender and cancer type and stage should be included in a preference model as they showed substantial differences in preferences for several attributes. Age should also be included in the preference model, given the large amount of prior literature on lung cancer (Blinman et al., 2010; Schmidt et al., 2016; Sugitani et al., 2020). Cancer stage, experience of surgery, experience of radiotherapy, ECOG PS, experience of anticancer drugs, and experience of adverse events of anticancer drugs are highly correlated; the inclusion of these factors in the model should be based on objectives of the study. Including cancer stage in a model would be useful because stage influences prognosis. ECOG PS reflects the patients’ living environment and may affect preferences of factors other than cancer stage. Annual income, education level, working status, dependents, and living situation were also highly correlated, and it is difficult to determine the one to be included in the model. Economic dependency status in a family may have an impact on the importance of survival, but education level and annual income are possible confounders, making it difficult to determine the primary factor. Education level has been noted to affect preferences in several lung cancer studies (Muhlbacher and Bethge 2015; Lehman et al., 2016). Concerning preferences for cost attributes such as drug prices, it would be appropriate to include annual income rather than education level, considering the strength of the correlation. Working status and living situation (i.e., whether someone lives with the patient) could influence the importance of adverse events, and the frequency and duration of administration could influence daily behaviors, but our results showed a limited impact of these parameters. It would be preferable to collect information on the types of anticancer drugs and specific adverse events previously experienced, as they seem to significantly affect our results. This information can also be used to infer preferences for drug use and other factors.

A large percentage of respondents had at least one or more “incorrect” choices for each of the four dominated pair questions; only 47.5% of respondents answered all of them correctly. The reliability of web-based conjoint analysis has been doubted (Melles et al., 2000). For example, a web-based survey of solid cancer patients in the US (Johnson et al., 2006) reported that 2.3% of respondents chose the same answer for all responses and 5.3% of respondents chose the wrong answer for the dominated pair questions. In this study, we found little difference in preference results after including and excluding respondents with “incorrect” choices. However, a slight simplification of the survey design could have reduced “incorrect” responses by lowering the respondent burden, especially for elderly patients with lung cancer. Although we reduced the number of attribute levels to examine more attributes than in other studies, the tradeoff between the number of attributes and levels may have resulted in a set of choices that were less psychologically burdensome for the respondents. Setting an appropriate reward level can also influence respondents’ incentives and thus the quality of responses. A low reward is likely to reduce the seriousness of the responses, while a high reward may attract unreliable respondents.

This study has several limitations. First, we could not assess the importance of attributes in different settings, such as thyroid dysfunction in immunotherapy or bleeding events in molecularly targeted drugs, or change attribute levels beyond our settings. Second, our respondent population may not be representative of the “average” patient. Compared to the most recent lung cancer statistics in Japan (Statistics Bureau of Japan 2019), the respondents in this study were younger, more likely to be male, and more likely to live in urban areas than the “average” patients. The importance of risk by conjoint logit model may be underestimated in this study because our results by the hierarchical Bayesian logit model show that the elderly and women are a more risk-important population, and the Japanese lung cancer patient population is older and includes more women than our sample. Third, as indicated in previous studies (Blinman et al., 2010; Schmidt et al., 2016; Sugitani et al., 2020), our study also suggested that demographic characteristics such as cancer stage and experience of anticancer drugs may influence patient preferences; therefore, it is inappropriate to apply just the conditional logit model that assumes a similar preference for all respondents. Besides, the results of the hierarchical Bayesian logit model and subpopulation analysis in our study should be interpreted with caution, as they were not designed in advance sufficient accuracy. Due to limited research and the difficulty in collecting a sufficient number of generalizable responses, the validity of a model with demographic characteristics cannot be guaranteed at this time.

## Conclusion

This is the first study to focus on discrete choice experiment to determine the preferences of Japanese patients with lung cancer. Survival time was the most important attribute, followed by adverse events, PFS, and frequency and duration of administration. The preferences were dependent on demographic characteristics (e.g., gender and age) and disease background (e.g., cancer type and stage). Interestingly, the experience of cancer drug therapies and adverse events seems to have a substantial impact on the preferences of hypothetical drug treatment. These results suggest the importance of patient experience considering patient preference. Hereafter, it would be better to design a quantitative preference study and analysis model that also takes into account the patient’s demographic and experience. Understanding patient preferences in detail would help select drugs that satisfy their needs and inform decisions for the clinical development of drugs for future patient generations.

## Data Availability

The datasets analyzed for this study are not publicly available because they contain information that could compromise respondents’ privacy and consent. Requests to access the datasets should be directed to YS, sugitaniyso@chugai-pharm.co.jp.
