# Supplementary material for: Patient Preferences for Attributes of Chemotherapy for Lung Cancer: Discrete Choice Experiment Study in Japan
Source: Front Pharmacol. 2021 Jul 20;12:697711. doi: 10.3389/fphar.2021.697711 (PMC8329447; doi:10.3389/fphar.2021.697711)
Supplement: Supplementary file 1 [file DataSheet1.pdf]

Supplementary Material

1 Supplementary Material 1: Formula used for data analysis

1.1 Conditional logit model

Utility function:

$$U_{in} = V_{in}(\beta_i, X_{in}) + \varepsilon_{in}$$

Choice Model:

$$P_n(i|J) = \frac{e^{V_{in}(\beta_i, X_{in})}}{\sum_{j=1}^{J}\left(e^{V_{jn}(\beta_j, X_{jn})}\right)}$$

1.2 Hierarchical bayes logit model

Utility function:

$$U_{in} = V_{in}(\beta_{in}, x_{in}) + \varepsilon_{in}$$

$$\beta_{in} = h_i + \sum_{k=1}^{K}(f_{ik} z_{nk}) + d_{in}$$

Choice Model:

$$P_n(i|J) = \frac{e^{V_{in}(\beta_{in}, X_{in})}}{\sum_{j=1}^{J}\left(e^{V_{jn}(\beta_{jn}, X_{jn})}\right)}$$

1.3 Explanations

n: one individual responder

i, j: one alternative from J

J: set of alternatives

U_{in}: Utility of alternative i for individual n

V: explainable component of utility

β: estimated coefficients

x: attribute level

ε: non-explainable or random component of utility

h: vector of estimated coefficients without effects of demographic parameters

k: one demographic parameter from K

K: set of demographic parameters

f_k: estimated coefficients effected by demographic parameter k

z: value of demographic parameter

d: random effect

2 Figures

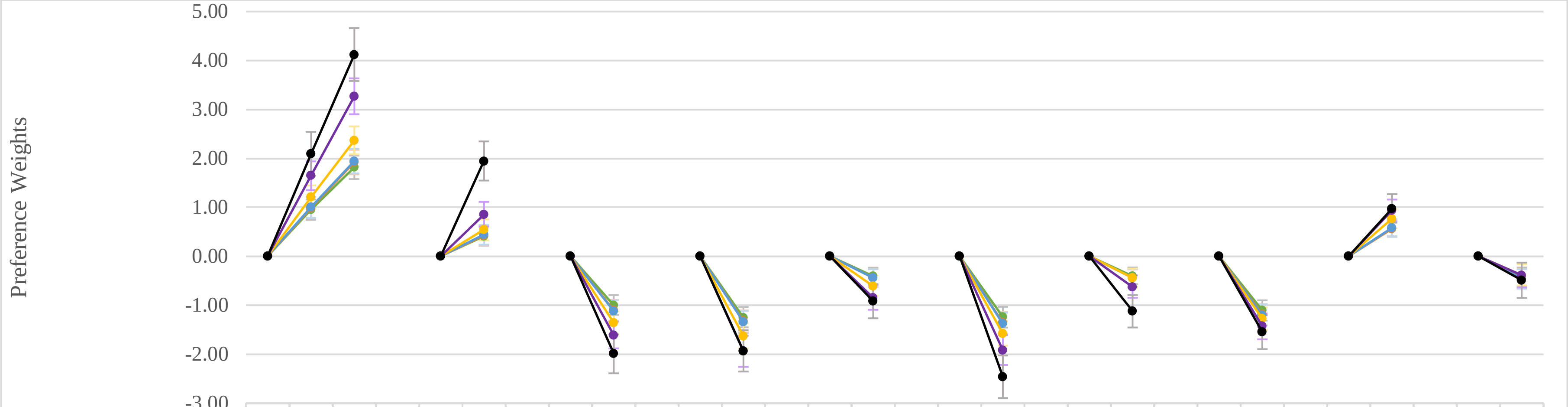

	OS			PFS		Diarrhea		Nausea		Fatigue		Rash		ILD		BMS		Freq of Admin		Duration of Admin	
	10 months (ref.)	15 months	20 months	4 months (ref.)	9 months	50% (ref.)	100%	10% (ref.)	50%	10% (ref.)	30%	10% (ref.)	80%	0% (ref.)	5%	0% (ref.)	20%	every week (ref.)	every 3 weeks	30 min (ref.)	180 min
All (N=200)	0.00	0.95	1.82	0.00	0.41	0.00	-0.9	0.00	-1.2	0.00	-0.4	0.00	-1.2	0.00	-0.4	0.00	-1.1	0.00	0.57	0.00	-0.4
Cohort A (N=191)	0.00	0.99	1.93	0.00	0.43	0.00	-1.1	0.00	-1.3	0.00	-0.4	0.00	-1.3	0.00	-0.4	0.00	-1.1	0.00	0.57	0.00	-0.4
Cohort B (N=190)	0.00	1.00	1.95	0.00	0.44	0.00	-1.1	0.00	-1.3	0.00	-0.4	0.00	-1.3	0.00	-0.4	0.00	-1.2	0.00	0.59	0.00	-0.4
Cohort C (N=177)	0.00	1.21	2.37	0.00	0.54	0.00	-1.3	0.00	-1.6	0.00	-0.6	0.00	-1.5	0.00	-0.4	0.00	-1.2	0.00	0.77	0.00	-0.3
Cohort D (N=148)	0.00	1.65	3.27	0.00	0.86	0.00	-1.6	0.00	-1.9	0.00	-0.8	0.00	-1.9	0.00	-0.6	0.00	-1.4	0.00	0.94	0.00	-0.3
Cohort E (N=95)	0.00	2.09	4.12	0.00	1.95	0.00	-1.9	0.00	-1.9	0.00	-0.9	0.00	-2.4	0.00	-1.1	0.00	-1.5	0.00	0.98	0.00	-0.4

Supplementary Material 2. Sensitivity analysis of conjoint analysis without low-reliability responders. Cohort A: population without respondent who chose the same option for all questions, Cohort B: population without respondent who choose the wrong answer in the dominated pair questions four times, Cohort C: population without respondent who choose the wrong answer in the dominated pair questions three times, Cohort D: population without respondent who choose the wrong answer in the dominated pair questions twice, Cohort E: population without respondent who choose the wrong answer in the dominated pair questions once. BMS: Bone Marrow Suppression, freq.: frequency, ILD: Interstitial Lung Disease, OS: Overall Survival, PFS: Progression Free Survival, ref.: reference.

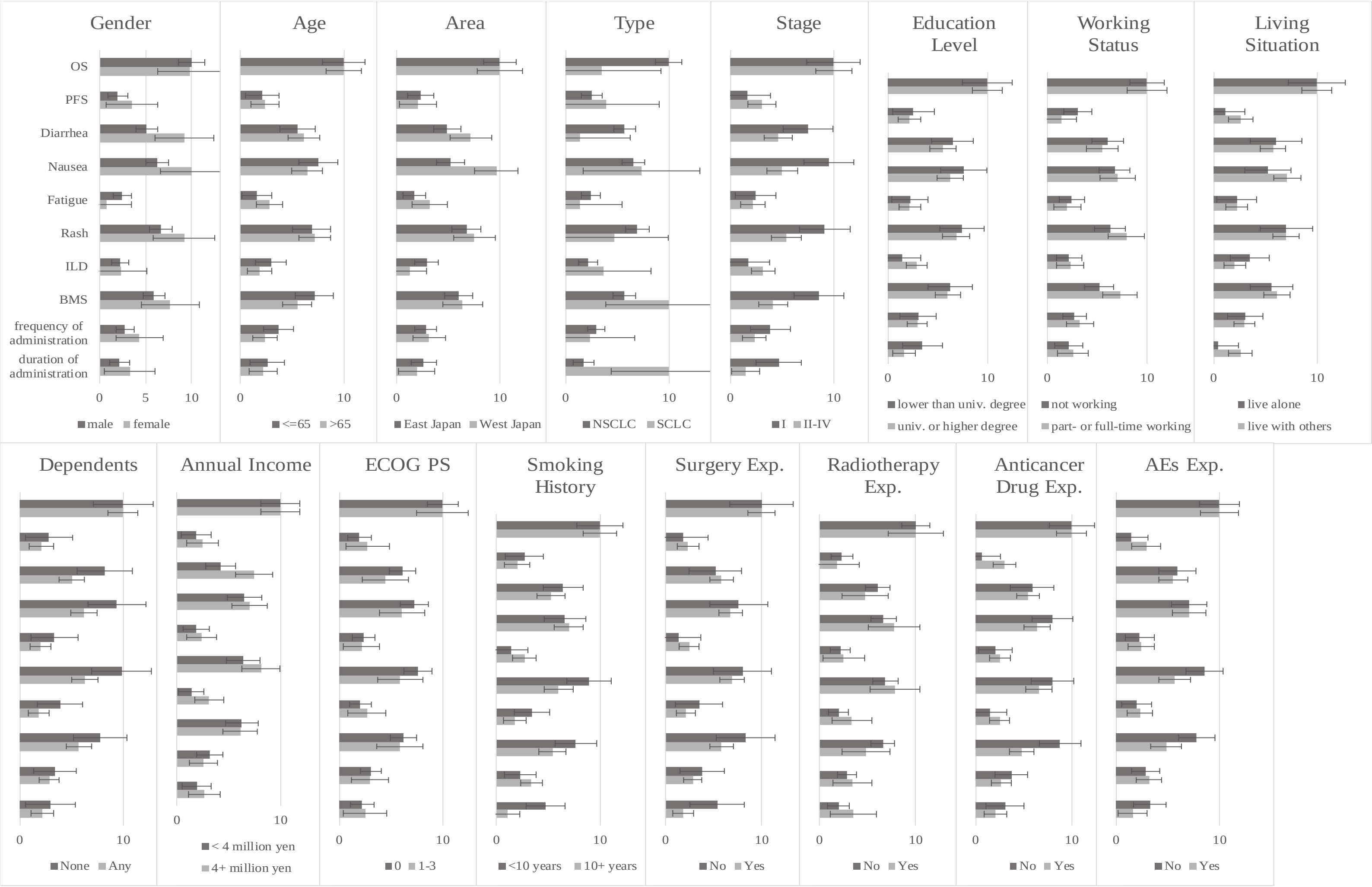

Supplementary Material 3. Standardized results of demographic subgroup analyses. BMS: Bone Marrow Suppression, ECOG PS: Eastern Cooperative Oncology Group performance status, exp.: experience, ILD: Interstitial Lung Disease, NSCLC: Non-Small-Cell Lung Cancer, OS: Overall Survival, PFS: Progression Free Survival, ref.: reference, SCLC: Small-Cell Lung Cancer, univ.: university.

	Gender	Age	Area	Type	Stage	Education Level	Working Status	Living Situation	Dependen ts	Annual Income	ECOG PS	Smoking History	Surgery Exp.	Ratiother apy Exp.	Anticance r Drug Exp.	AEs Exp.
Gender	-	0.0103	0.2461	0.9342	0.6494	0.0003	0.0075	0.0036	<.0001	0.0734	0.9259	<.0001	0.0949	0.1288	0.0569	0.3455
Age		-	0.4819	0.7053	0.0456	0.3163	<.0001	0.5706	0.4414	0.0665	0.1965	0.0072	0.009	0.1268	0.2231	0.1489
Area			-	0.2439	0.6243	0.9889	0.8285	0.2681	0.4821	0.869	0.6576	0.7536	0.7001	0.5507	0.0844	0.0566
Type				-	0.3564	0.0119	0.8687	0.7555	0.1591	0.4629	0.3361	0.5472	0.5678	0.4419	0.6858	0.4212
Stage					-	0.7182	0.4294	0.5486	0.9667	0.5047	0.0003	0.1711	<.0001	<.0001	<.0001	<.0001
Education Level						-	0.5907	0.6951	0.0397	0.7039	0.002	0.1763	0.2018	0.9485	0.9578	0.5955
Working Status							-	0.8387	0.0739	<.0001	0.0476	0.7337	0.8478	0.0155	0.7222	0.0886
Living Situation								-	<.0001	0.1184	0.9668	0.5573	0.2051	0.986	0.7344	0.3115
Dependents									-	0.0006	0.7243	0.0816	0.0301	0.9823	0.6559	0.7402
Annual Income										-	0.523	0.3252	0.5769	0.447	0.3234	0.2746
ECOG PS											-	0.0663	0.1278	0.0104	0.0141	0.0055
Smoking History												-	0.5847	0.2037	0.0344	0.0641
Surgery Exp.													-	<.0001	0.0015	<.0001
Ratiotherapy Exp.														-	<.0001	<.0001
Anticancer Drug Exp.															-	<.0001
AEs Exp.																-

Supplementary Material 4. P-value by chi-squared test between demographic parameters. ECOG PS: Eastern Cooperative Oncology Group performance status, exp.: experience.

	Intercept	Gender (ref. male)	Age (ref. 30-39)	Area (ref. East Japan)	Type (ref. NSCLC)	Stage (ref. I)	Education level (ref. lower than univ. degree)	Working status (ref. not working)	Annual income (ref. less than 4 million yen)	Exp. of anticancer drug (ref. No)	Exp. of AEs by anticancer drug (ref. No)
		female	per 10 years old	West Japan	SCLC	II-IV	univ. or higher degree	Part- or full-time working	4 million yen or more	Yes	Yes
OS (ref. 10 months)											
15months	-0.02	0.54	1.09 +	2.73 ++	-1.80	2.78 +	2.64 ++	1.97	-1.17	-0.85	3.84 +
20months	-0.15	2.49	3.24 ++	2.43	4.06 +	9.19 ++	5.07 ++	3.86 +	1.69	-3.12	0.74
PFS (ref. 4 months)											
9 months	-0.55	0.25	0.15	1.02	-2.98 -	3.93 ++	0.79	0.65	1.00	0.36	-1.75
Diarrhea (ref. 50%)											
100%	0.26	-3.75 --	-1.00	-4.70 --	3.72 +	-0.62	-2.19 -	-5.44 --	1.53	-0.85	-0.41
Nausea (ref. 10%)											
50%	-0.01	-7.22 --	-1.81 --	-5.52 --	2.30	1.26	-2.34 -	-2.17	-0.29	-0.55	-2.05
Fatigue (ref. 10%)											
30%	0.09	1.11	-1.41 --	-3.15 --	5.19 ++	-1.97	0.08	-2.08	-0.23	-0.09	0.05
Rash (ref. 10%)											
80%	-0.26	-3.05	-3.34 --	-0.26	1.33	-3.39 -	-2.31 -	-3.15 -	0.75	-3.99 -	5.60 ++
ILD (ref. 0%)											
5%	-0.19	2.20 +	0.11	0.94	-2.08	-3.38 --	-1.81 -	-1.98 -	1.15	-0.29	3.32 +
BMS (ref. 0%)											
20%	-0.36	-4.90 --	-1.27 -	-0.75	-1.44	-0.79	-2.84 --	-4.18 --	0.35	-0.60	1.91
Frequency of Administration (ref. every week)											
every 3 weeks	0.29	3.25 +	1.49 ++	1.38	1.75	0.76	1.64 +	0.91	0.15	-3.41 -	2.86 +
Duration of administration (ref. 30 minutes)											
180 mintues	0.26	-0.94	-1.39 -	3.34 ++	-2.99	0.25	1.04	1.44	0.51	-3.21 -	3.79 +

Supplementary Material 5. Effect for preferences of each attributes by demographic parameters without imputation. BMS: Bone Marrow Suppression, exp.: experience, ILD: Interstitial Lung Disease, NSCLC: Non-Small-Cell Lung Cancer, OS: Overall Survival, PFS: Progression Free Survival, ref.: reference, SCLC: Small-Cell Lung Cancer, univ.: university.
